# Editorial: Exosomes in cardiovascular diseases: Mechanism, diagnosis, and therapy

**DOI:** 10.3389/fcvm.2022.1018381

**Published:** 2022-10-20

**Authors:** Xiao Zhang, Mengting Zeng, Yuting Liu, Hongyun Wang, Yunlong Huang, Junjie Xiao

**Affiliations:** ^1^Institute of Geriatrics, Affiliated Nantong Hospital of Shanghai University (The Sixth People's Hospital of Nantong), School of Medicine, Shanghai University, Nantong, China; ^2^Cardiac Regeneration and Ageing Lab, Institute of Cardiovascular Sciences, Shanghai Engineering Research Center of Organ Repair, School of Life Science, Shanghai University, Shanghai, China; ^3^Department of Pharmacology and Experimental Neuroscience, University of Nebraska Medical Center, Omaha, NE, United States

**Keywords:** exosomes, cardiovascular diseases, diagnosis, therapy, diagnose, extracellular vesicles

Among non-communicable diseases, cardiovascular disease (CVD) is the leading cause of mortality worldwide, which is associated with increased morbidity and hospitalization (1). To date, few effective strategies have been developed to cure CVD including heart failure and ischemic-reperfusion injury, etc. Therefore, exploring effective therapeutical strategies is essential to CVD treatment. In recent years, extracellular vesicles (EV) have attracted growing attention in the diagnosis and treatment of CVD (2, 3) due to their specific characteristics, including excellent biocompatibility and low immunogenicity (4). This Research Topic aims to provide more constructive scientific findings and new horizons in CVD diagnosis and treatment, which may help promote future clinical trials of EV in treating CVD.

In this issue, Ma et al. creatively conduct an overview of EV research in CVD *via* a bibliometric analysis. The authors systematically collect the last 20 years of research on Web of Science Core Collection and perform a bibliometric analysis with visual tools (Citespace and Vosviewer). The results demonstrate that increasing attention was significantly paid to the capacity of EV in CVD from 2017 onwards, indicating the increasing popularity of the subject in this field. Furthermore, the authors conclude most of these studies focused on EV as biomarkers for CVD diagnosis, delivery vehicles, and a potential strategy for treating myocardial infarction.

The study of Yao et al. prospectively performs atrial fibrillation (AF) modeling in Canines and investigates the function of EV in AF development. The authors reveal that blocking the release of small EV by GW4869 could alleviate AF by reducing atrial fibrosis. Mechanistically, EV-enclosed miR-21-5p targets the downstream TIMP3/TGF-β1 pathway and induced fibrosis. Inhibiting the release of EV may be a potential strategy for AF treatment, which may help accelerate new clinical trials of AF treatment. Chen et al. systematically review the role of EV and EV-enclosed non-coding RNAs (NcRNAs) in the diagnosis and treatment of AF in more detail. Notably, EV-enclosed NcRNAs may also play a crucial role in the progression of AF.

The EV-mediated interaction between adipose tissue and blood vessels may play an important role in CVD. In this Research Topic, Liu et al. and Yang et al. explore the role of adipose-derived EV in lipid metabolism, which is closely associated with vascular homeostasis. Epicardial adipose tissue (EAT)-derived EV-enclosed miR-3064-5p is identified as a key molecule in regulating lipogenic differentiation.

Interestingly, EV is proven to be involved in air-pollution-associated cardiac injury. Hu et al. investigate the crosstalk between macrophages and cardiomyocytes. The authors reveal that ambient particulate matter could promote the release of EV and subsequently activate macrophages. EV-enclosed TGF-β derived from macrophages promotes the fibrotic alteration of cardiomyocytes, ultimately leading to cardiac dysfunction. This study sheds light on the underlying mechanism and potential therapeutical strategy for air pollution-associated cardiovascular disorders.

Notably, increasing evidence shows that platelet-derived EV are a crucial component of circulating nanoparticles in blood, indicating that platelet-derived EV play important roles in multiple pathological processes. In this Research Topic, Wei et al. summarize the role of platelet-derived EV in mediating intercellular communication, which contributes to arterial thrombosis. This review provides a new horizon as platelet-derived EV could provide promising biomarkers for the diagnosis of CVD.

Overall, this Research Topic provides a relatively comprehensive understanding of the role and potential application of EV in CVD, including a bibliometric analysis, overviews, and some surprising experimental articles. These studies identify a novel cargo and signaling axis (EV-miR21-TIMP3/TGF-β1, miR-3064-5) in the development of CVD and provide a novel way of using different sources of EV and EV-enclosed cargo in diagnosing CVD. Nevertheless, EV-associated basic, translational, and clinical studies are still on the way. Here we sincerely hope that this Research Topic can provide readers with different viewpoints and new horizons in EV & CVD diagnosis and treatment, inspiring future studies on the therapeutical application of EV. In addition, we also hope the work can help to stimulate novel ideas in associated fields and improve the research progression of CVD.

## Author contributions

HW, YH, and JX are the topic editors of this issue and they have contributed to the writing and revising of the article. XZ, MZ, and YL contributed to drafting the Editorial. All authors approved it for publication.

## Funding

This work was supported by grants from the National Key Research and Development Project (2018YFE0113500 to JX), the National Natural Science Foundation of China (82020108002 and 81911540486 to JX and 82000253 to HW), the grant from Science and Technology Commission of Shanghai Municipality (20DZ2255400 and 21XD1421300 to JX), the Dawn Program of Shanghai Education Commission (19SG34 to JX), the Sailing Program from Science and Technology Commission of Shanghai (20YF1414000 to HW), Chenguang Program of Shanghai Education Development Foundation, and Shanghai Municipal Education Commission (20CG46 to HW).

## Conflict of interest

The authors declare that the research was conducted in the absence of any commercial or financial relationships that could be construed as a potential conflict of interest.

## Publisher's note

All claims expressed in this article are solely those of the authors and do not necessarily represent those of their affiliated organizations, or those of the publisher, the editors and the reviewers. Any product that may be evaluated in this article, or claim that may be made by its manufacturer, is not guaranteed or endorsed by the publisher.
